# Understanding Caregivers’ Influence on Preschoolers’ Eating Behaviors: An Integrative Review Guided by the Theory of Planned Behavior

**DOI:** 10.3390/children12020163

**Published:** 2025-01-29

**Authors:** Qutaibah Oudat, Elaine L. Miller, Sarah C. Couch, Rebecca C. Lee, Tamilyn Bakas

**Affiliations:** 1 Department of Population Health, College of Nursing, University of Cincinnati, Cincinnati, OH 45221, USA; millerel@ucmail.uc.edu (E.L.M.); lee2rc@ucmail.uc.edu (R.C.L.); bakastn@ucmail.uc.edu (T.B.); 2 Department of Rehabilitation, College of Allied Health Sciences, University of Cincinnati, Cincinnati, OH 45221, USA; couchsc@ucmail.uc.edu

**Keywords:** preschool children, dietary behaviors, feeding practices, caregivers, parental influence

## Abstract

Background/Objectives: Primary caregivers of children play a significant role in developing their dietary behaviors. Guided by the Theory of Planned Behavior (TPB), this integrative review aimed to synthesize studies examining how personal and household characteristics, caregivers’ dietary beliefs, intentions, and feeding practices influence the eating behaviors of preschool-aged children (2–5 years). Methods: PubMed, CINAHL, and PsycINFO databases were searched for peer-reviewed articles published between January 2014 and September 2024. The expanded PRISMA 2020 checklist was used to guide the literature search and report the results. The Johns Hopkins Nursing Evidence-Based Practice (JHNEBP) was also used to evaluate the quality of the selected articles. Results: A total of 10 studies were included in the final analysis. The studies revealed that preschoolers’ eating behaviors were significantly influenced by personal characteristics (e.g., caregivers’ BMI, and weight perceptions) and household factors (e.g., food availability). Additionally, caregivers’ feeding practices, such as restriction, modeling, and permissiveness, played a pivotal role in shaping children’s eating habits. Although caregivers expressed intentions to provide a healthy diet, they often faced barriers, including conflicting work schedules, financial constraints, and logistical challenges, which impeded their ability to consistently promote healthy eating behaviors. Conclusions: Caregivers’ beliefs, intentions, and feeding practices are pivotal in shaping preschoolers’ eating behaviors. However, the limited available literature and the underexplored mechanisms linking these factors make it challenging to draw solid conclusions. Future research should address these gaps and consider integrating caregiver-focused factors into tailored interventions to promote healthier eating habits in preschool-aged children. This can aid healthcare professionals in designing culturally and contextually sensitive strategies for improving childhood nutrition.

## 1. Introduction

Eating behaviors develop during the early years of life through a combination of biological and behavioral processes [1,2]. The preschool years, in particular, are critical for shaping and solidifying dietary patterns that can continue into adulthood and affect long-term health outcomes [3,4]. During the preschool years, children learn when, where, and how to eat through direct and indirect interactions with their surrounding environment, especially their primary caregivers [5].

Existing reviews have used various theoretical frameworks to explain the influence of familial and social determinants on the eating behaviors of preschoolers. For example, a narrative review by Mahmood et al. (2021) [3] utilized a conceptual model derived from the Social Ecological Model (SEM) to understand the impact of parental dietary habits on the eating habits of their preschool children (ages 2–5 years). The findings showed that various home- and family-related determinants, such as parental eating habits and feeding practices of caregivers, were significantly affecting the eating habits of preschoolers. Feeding practices, such as monitoring, covert restriction, and modeling healthy eating were found to significantly promote healthy eating. In contrast, overt restriction, persuasive feeding, and the use of rewards for eating and behavior were significantly associated with poor eating behaviors [3]. Additionally, Scaglioni et al. (2018) [6] used the Ecological Systems Theory (EST) to narratively explain the interaction between the eating behaviors of children (ages 6 months through 19 years) and various potential factors. The review has demonstrated a consistent paramount influence of family dynamics, parental practices, and broader environmental factors on the eating behaviors of children [6]. Interestingly, another review used the Family-Centered Framework to explore the existing evidence on paternal influences on a child’s dietary intake (birth to 18 years old) [7]. The review has revealed that fathers can affect their children in several ways, such as dietary behaviors and feeding practices. Furthermore, another review by Rieble et al. (2015) [8] utilized the constructs of the Theory of Planned Behavior (TPB) to predict and understand nutrition-related behaviors in youth (ages 2–18). The majority of the participants in the included studies had an average age of around 8 to 12 years. The findings indicated that TPB is a promising framework for understanding and predicting nutrition-related behaviors in youth. Attitudes and intentions were significant predictors of actual dietary behavior [8]. While these reviews included children and adolescents aged 2 to 18 years, fewer studies in these reviews focused on the younger age group of preschoolers (2–5 years). Additionally, the mechanisms through which the caregiver’s beliefs and intentions translate into children’s actual dietary behaviors were not fully explained. This made it difficult to draw firm conclusions about dietary behaviors and interventions for younger children specifically.

**Figure 1:**
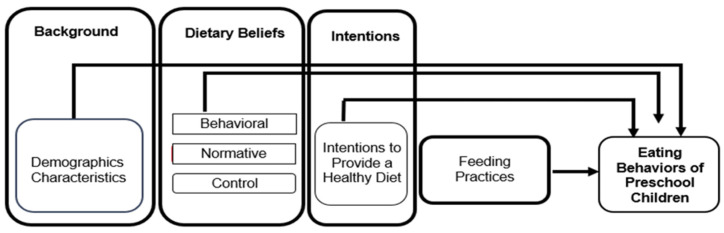
Conceptual model to guide this review.

The TPB provides a comprehensive framework for examining the determinants of health-related behaviors by considering the interplay between beliefs (behavioral, normative, and control), intentions, and actual behavior [9]. The constructs of the TPB have been widely used to explain various health behaviors, including smoking and condom use, demonstrating its effectiveness in understanding a broad spectrum of health-related behaviors [10]. When applied to the relationship between caregivers and preschoolers’ eating behaviors, we propose that the TPB can explain how caregivers’ dietary beliefs and intentions translate into specific feeding practices, thereby influencing children’s dietary patterns. Understanding the impact of caregivers’ beliefs and intentions, and feeding practices, along with personal and household characteristics can aid healthcare providers (e.g., nurses) and scientists to tailor educational and counseling strategies to address potential barriers and facilitate healthy eating behaviors among preschoolers. Guided by a framework derived from the TPB, the purpose of this integrative review was to systematically synthesize the existing literature on how personal and household characteristics, caregivers’ dietary beliefs and intentions, and feeding practices influence preschoolers’ eating behaviors. See the conceptual model presented in Figure 1.

## 2. Materials and Methods

We used the expanded PRISMA 2020 checklist [11] to guide our literature search and report the results. This checklist facilitated the documentation of each phase of the search process, including the eligibility criteria and quality assessment methods.

### 2.1. Information Source, Search Strategy, and Queries

In this integrative review, the primary outcome of interest was the eating behaviors of preschool-aged children. We aimed to examine how this outcome is influenced by predictors such as parental dietary beliefs, intentions to provide a healthy diet, and feeding practices of primary caregivers. In our review, we defined “primary caregiver” as the individual responsible for raising children aged 2 to 5 years, including those who spend time with the child, supervise activities, and manage meals, such as parents, grandparents, guardians, or older siblings.

A literature search in three electronic databases, PubMed, CINAHL Plus with Full Text, and APA PsycINFO (via EBSCOhost), was used to find eligible articles published between January 2014 and September 2024. This time frame enables us to determine all of the relevant articles in the past decade, thus providing more recent findings. Our search strategy was crafted and guided by the focus of the review and the constructs of the TPB. We used a combination of keywords and synonyms to effectively capture the relevant literature. Primary outcome terms included “children’s eating behaviors” and “preschoolers”. For predictors, we incorporated terms related to parental roles (e.g., “parents”, “caregivers”), feeding practices, and TPB constructs like “beliefs”, “attitudes”, and “intentions”. Additionally, we included terms reflecting personal and home characteristics affecting children’s eating behaviors, such as “socioeconomic status”, “parental education”, and “home environment”. Boolean operators and database filters refined the search results to ensure a comprehensive retrieval of the pertinent studies.

In PubMed, the search query focused on identifying articles related to children’s eating behaviors, parental or caregiver roles, and feeding practices. The query was as follows:

“(children eating behaviors) AND (parents OR caregivers OR mother OR father OR parent OR guardians) AND (“feeding practices” OR “feeding strategies” OR “dietary behaviors”) AND (beliefs OR perceptions OR views OR attitudes OR opinions OR knowledge OR “behavioral beliefs” OR “normative beliefs” OR “control beliefs” OR intentions OR “decision-making”) AND (“socioeconomic status” OR “parental education” OR “home environment” OR age OR sex OR gender OR race OR ethnicity OR “cultural background” OR BMI OR “weight perceptions” OR “concerns about child weight” OR “household factors” OR “food availability” OR “screen time” OR “TV viewing”).

Similarly, in the CINAHL Plus with Full Text and APA PsycINFO databases (via EBSCOhost), a tailored query was employed to account for database-specific indexing and search capabilities. The search terms used were as follows:

“children eating behaviors AND (parents OR caregivers OR mother OR father OR parent OR guardians OR guardian) AND feeding practices OR children eating behaviors AND (parents OR caregivers OR mother OR father OR parent OR guardians OR guardian) AND (beliefs OR perceptions OR views OR attitudes OR opinions OR knowledge) OR children eating behaviors AND (parents OR caregivers OR mother OR father OR parent OR guardians OR guardian) AND behavioral beliefs OR children eating behaviors AND (parents OR caregivers OR mother OR father OR parent OR guardians OR guardian) AND normative beliefs OR children eating behaviors AND (parents OR caregivers OR mother OR father OR parent OR guardians OR guardian) AND control beliefs OR children eating behaviors AND (parents OR caregivers OR mother OR father OR parent OR guardians OR guardian) AND intentions OR children eating behaviors AND (parents OR caregivers OR mother OR father OR parent OR guardians OR guardian) AND (BMI OR “weight perceptions” OR “concerns about child weight” OR household OR “food availability” OR “screen time” OR “TV viewing”).

We applied filters to ensure that the search results were relevant, focused, and manageable. Specifically, we included studies that were available in free full text or full text, written in English, focused on preschool children aged 2–5 years, and published between January 2014 and September 2024.

### 2.2. Inclusion/Exclusion Criteria

Potential articles were included if they fulfilled the eligibility criteria. Our eligibility criteria included the following: (1) studies that used either qualitative or quantitative research designs, (2) written in English, (3) available in full text, and (4) studies that involved at least one primary caregiver and preschool-aged child. Additionally, studies were included if they examined the association between the eating behaviors of preschoolers and one or more of the following predictors: personal and household characteristics, primary caregiver dietary beliefs, intentions to provide a healthy diet, or feeding practices. We excluded studies that involved children or caregivers with medical conditions or nutritional disorders, drug trials, and non-original research (such as books, abstracts, conference proceedings, dissertations, theses, expert opinions, unpublished manuscripts, and newspaper articles).

### 2.3. Selection Process

**Figure 2:**
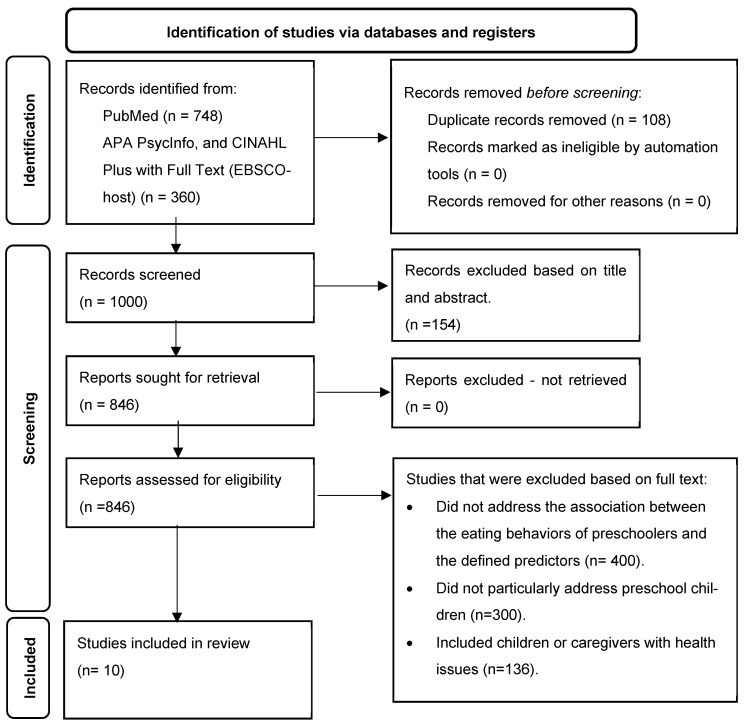
PRISMA flow diagram.

The PRISMA flow diagram (Figure 2) was followed to organize the selection process [11]. Initially, 1108 studies were identified through our literature search and imported into EndNote 20, a citation management software that facilitates collaboration by providing a remote platform for managing references and identifying eligible studies [12]. After removing duplicates (*n* = 108), 1000 studies remained for screening. Two independent reviewers (QO and EM) independently screened each record at the title and abstract and selected full-text articles for eligibility based on the predefined inclusion and exclusion criteria. Any disagreements between reviewers were resolved through discussion, and when necessary, a third reviewer (TB) was consulted to reach a consensus. No automation tools were used in the selection process. The review team confirmed the final selected studies to ensure adherence to the inclusion criteria.

### 2.4. Data Extraction and Evaluation

Following the guidelines outlined by Younas and Ali (2021) [13], we extracted data to develop comprehensive summary tables that facilitated the comparison of the reviewed articles (see Supplementary Table S1). This table includes the following: (a) detailed information, such as authors, country of study, research purpose, design, conceptual framework, sample characteristics, data collection methods, concepts and measures, key findings, and conceptual contributions; and (b) assessments of each study’s strengths and limitations using critique guidelines.

To systematically evaluate the strength and reliability of the evidence in each selected article, we used the Johns Hopkins Nursing Evidence-Based Practice (JHNEBP) appraisal tool. This tool categorizes evidence into five levels based on the study design and methodological rigor [14]. The level of evidence (LOE) for each study was presented in an additional column in the comprehensive table. See Supplementary Table S1, which contains a summary of the research findings associated with the eating behaviors of preschool children.

## 3. Results

A total of 1000 studies were included in the screening. Out of these, 154 studies were excluded after screening their titles and abstracts for not fulfilling the inclusion criteria, and the full texts of four additional articles were not available in the databases searched. This left 846 full-text articles to be retrieved and thoroughly reviewed according to our inclusion and exclusion criteria. Following the full-text screening, 836 articles were excluded for several reasons, including studies that (a) did not address the association between the eating behaviors of preschoolers and the defined predictors (*n* = 400), (b) did not specifically focus on preschool children (*n* = 300), and (c) included children or caregivers with health issues (*n* = 136). In total, 10 articles fulfilled the inclusion criteria and were included in the final analysis.

### 3.1. Study Design and Samples

Of the ten studies included in this review, two were qualitative studies [15,16] that explored how caregivers’ beliefs, parenting styles, and practices affect the eating behaviors of children aged between 2 and 5 years. These qualitative studies had sample sizes ranging from 21 to 33 participants, with the caregivers typically being in their 30s. Additionally, eight studies were quantitative, comprising one longitudinal observational study [17] and seven cross-sectional studies [18,19,20,21,22,23,24]. These studies investigated the relationships between various parental factors, such as home environmental characteristics, feeding practices, weight perception, and children’s eating behaviors. In the quantitative studies, the sample sizes ranged from 115 to 1616 caregiver–child dyads. The caregivers were typically in their late 20s to early 40s, and children were generally between 2 and 5 years old. Overall, all of the studies included in this review were nonexperimental (level III) based on the JHNEBP appraisal tool.

Most studies predominantly involved mothers as the caregivers, with a significant portion holding higher education levels. This trend was consistent across different countries and cultural contexts. The sample populations were diverse, encompassing various ethnic groups, including Hispanic, White/Caucasian, Chinese, Brazilian, Ethiopian, and Saudi Arabian, and a range of socioeconomic backgrounds. This diversity provided a comprehensive context for examining parental feeding practices and child eating behaviors among preschool-aged children. The studies were conducted in a variety of countries, enhancing the cross-cultural relevance of the findings. The locations included the United States [15,16,17], Ireland [18], UK [19], Singapore [20], Ethiopia [21], China [22], Brazil [23], and Saudi Arabia [24]

### 3.2. Measures to Evaluate the Eating Behaviors of Children

The studies reviewed collected data on children’s eating behaviors through self-report questionnaires completed by the primary caregivers/parents of young children. One of the most frequently used tools across these studies was the Children’s Eating Behavior Questionnaire (CEBQ) (35 items, eight subscales) [25]. This was utilized in several studies, including those by Berge et al. (2020) [17], Haycraft et al. (2017) [19], Gebru et al. (2021) [21], and Kutbi and Mosli (2024) [24]. The CEBQ is designed to evaluate multiple dimensions of children’s eating behaviors, such as food responsiveness, satiety responsiveness, enjoyment of food, emotional overeating, and food fussiness.

Additionally, three studies [20,22,23] used three different versions of the Food Frequency Questionnaires (FFQs) [26,27,28] to collect data on the preschooler’s habitual intake. Furthermore, only one study by Bassul et al. (2020) [18] used markers of a healthy (fruit and vegetables) or unhealthy (confectionary/SSBs) diet [29] to assess the types and frequencies of foods consumed.

### 3.3. Personal and Household Characteristics, and Eating Behaviors of Preschoolers

Personal Characteristics. Overall, the study results found that personal characteristics, such as caregivers’ weight perception, concerns about child weight, and BMI, can significantly influence preschoolers’ eating behaviors. However, few studies in this review have directly addressed these factors.

In China, Xiang et al. (2021) [22] examined how parental perceptions of child weight and related concerns impact feeding practices and dietary intake among preschoolers. The study found that parents who underestimated their child’s weight were more likely to apply pressure to eat, especially if the child was of normal weight or overweight/obese. Conversely, overestimating a child’s weight was linked to higher traditional dietary pattern scores, particularly among underweight children. The traditional dietary pattern, specific to Chinese culture, is characterized by a high intake of grains, roots and tubers, legumes, nuts, eggs, livestock and poultry meat, fish or shellfish, and Vitamin A-rich fruits and vegetables. This reflects the nutrient-dense and balanced food choices typical of traditional Chinese cuisine. Parents concerned about their children becoming overweight imposed greater food restrictions on normal-weight children. Additionally, preschoolers whose caregivers desired them to be thinner and worried about potential overweight or obesity were more likely to consume more snacks [22]. Another study in Saudi Arabia by Kutbi and Mosli (2024) [24] investigated the relationship between maternal concerns and perceptions of child weight and children’s eating behaviors. Their findings indicated that the maternal concern about a child’s weight was positively correlated with the child’s enjoyment of food and responsiveness to food cues. However, this concern was not significantly associated with the parent’s feeding practices, particularly the restriction on eating and using food as a reward. In contrast, the maternal perception of the child’s risk of becoming overweight was inversely associated with the child’s slowness in eating. These results suggest that parental misperceptions and concerns can significantly impact children’s eating behaviors [24].

Caregivers’ BMI also significantly affects how they perceive their children’s eating behaviors. Haycraft et al. (2017) [19] found that mothers who were overweight or obese tended to view their children’s eating habits differently compared to mothers of a healthy weight. Specifically, overweight or obese mothers were more likely to believe that their children had a higher desire for drinks, particularly sugary or calorie-dense beverages. Additionally, mothers with higher BMIs were more likely to (a) give their child more control over eating, (b) encourage less balance and variety in the diet, (c) create a less healthy home food environment, and (d) model less healthy eating behaviors [19]. Similarly, Bassul et al. (2020) also found that a higher parental BMI was associated with a lower fruit and vegetable intake and a higher consumption of sweets and sugar-sweetened beverages (SSBs) among children. The study revealed that higher parental education was linked to higher fruit and vegetable consumption in children [18].

Household Characteristics. Household factors can also play a significant role in shaping children’s eating behaviors. Only one study in this review specifically addressed the impact of household characteristics, such as TV viewing and food availability, on children’s eating behaviors. Bassul et al. (2020) [18] examined how TV viewing and food availability and accessibility influence the eating behaviors of preschoolers. The study found that children who ate snacks while watching TV were 71% less likely to consume vegetables on a daily basis compared to those who did not. Additionally, watching TV for more than 1 h per day was associated with a lower vegetable intake and a higher intake of confectionery and sugar-sweetened beverages (SSBs). The study also highlighted that the availability of healthy foods and snacks plays a crucial role in promoting healthy eating habits. In contrast, the presence of sugary snacks and SSBs was positively linked to an increased consumption of these items among children [18]. However, the association between the eating behaviors of preschoolers and other household characteristics, such as sex, gender, race/ethnicity, and socioeconomic status, were not addressed in the studies reviewed.

### 3.4. Dietary Beliefs, Intention to Provide a Healthy Diet, and Eating Behaviors of Preschoolers

Behavioral Beliefs. Behavioral beliefs (BBs) refer to parents’ perceptions of the likely outcomes of certain dietary behaviors and the value they place on these outcomes. These beliefs influence how parents perceive healthy eating and its importance for their children’s health. In Latino immigrants living in the US, a study by Lindsay et al. (2018) showed that caregivers were aware of the importance of healthy eating for their children’s growth and development [16]. However, it is still unclear how these behaviors affect the overall dietary behaviors of children. The studies reviewed were limited in investigating the association between BBs and the eating behaviors of children.

Normative Beliefs. Normative beliefs (NBs) involve parents’ perceptions of social pressures and expectations regarding feeding practices and dietary habits. Among the reviewed studies, Lindsay et al. (2020) [15] explored the perspectives and practices of Brazilian immigrants regarding their children’s eating behaviors and feeding habits. The results showed that shared meals were essential for teaching children about healthy eating and Brazilian customs. Despite the strong normative beliefs emphasizing traditional and healthy foods, caregivers, particularly fathers, still permitted indulgence in sweets and high-calorie food [15]. This behavior may be linked to a lack of nutritional knowledge to balance cultural traditions with the realities of living in a new environment. However, this review has shown a lack of quantitative analysis on the direct effects of NBs on parental feeding attitudes and their subsequent influence on children’s eating behaviors.

Control Beliefs. Control beliefs relate to parents’ perceptions of the factors that facilitate or hinder their ability to provide a healthy diet, including resources, time, and self-efficacy. Lindsay et al. (2018) [16] highlighted that despite low-income Latino parents recognizing the importance of healthy eating, they faced several barriers that hindered their ability to provide a healthy diet for their children. These challenges were time constraints, neighborhood safety, the ability to keep up with their child, caring for more than one child, and financial limitations [16]. However, the studies in this review have not yet explored how these factors impact children’s eating behaviors.

Intentions to Provide a Healthy Diet. Intentions represent the motivational factors that influence behavior. It indicates the willingness of people to try and how much effort they plan to exert to perform the behavior. Caregivers with strong intentions, shaped by positive beliefs and social support, are more likely to adopt feeding practices that promote healthy eating behaviors. In this review, Lindsay et al. (2018) [16] pointed out that low-income Latino immigrant parents living in the United States aimed to model healthy eating and provide nutritious diets for their children but faced barriers such as financial constraints, conflicting work schedules, and neighborhood safety concerns [16]. Similarly, Lindsay et al. (2020) [15] reported that Brazilian immigrant fathers residing in the United States intended to provide healthy food and teach their children about nutrition but faced cultural and logistical challenges in doing so consistently. However, the studies reviewed did not examine the relationship between caregivers’ intentions and children’s eating behaviors.

### 3.5. Feeding Practices Associated with the Eating Behaviors of Preschoolers

Feeding practices are a set of goal-directed strategies used by primary caregivers to control or modify the eating and diet of their children [30]. Several feeding practices were identified in this review: (a) restriction, (b) pressure to eat, (c) instrumental feeding (using food as a reward), (d), modeling, teaching, and encouragement of healthy eating, (e) monitoring, (f) emotion feeding practice, and (g) permissive feeding practices.

Restriction. Restrictive feeding practices involve limiting children’s access to certain foods, often to control their weight or encourage healthier eating. Parental food restriction was found to influence children’s eating behaviors in various ways across different studies. Restrictive feeding practices showed mixed outcomes in influencing children’s eating behaviors. In Brazil, restricting food for weight control was shown to be more commonly used by caregivers with lower educational levels and higher concerns about their child’s overweight status. However, the study did report any associations between restricted feeding for weight control and the eating behaviors of children in the final multiple regression analysis [23]. On the other hand, in Ethiopia, food restriction was associated with increased food responsiveness, emotional overeating, and enjoyment of food among children, while negatively correlating with food fussiness and positively with emotional undereating [21]. In Singapore and China, restriction was linked to increased fruit intake and reduced snacking behaviors, respectively [20,22]. These discrepancies may stem from cultural differences, socioeconomic factors, or variations in how restriction is implemented. The findings suggest that while restriction can sometimes promote healthier eating, it may also lead to unintended negative consequences like increased overeating or emotional eating.

Pressure to Eat. The pressure to eat involves urging or forcing children to consume more food, often out of concern that they are not eating enough. Warkentin et al. (2018) [23] found that caregivers with a lower BMI and high concerns about a child’s weight were more likely to pressure their children to eat. Generally, the practice of pressuring children to eat was generally associated with negative outcomes or no significant effects [23]. While some studies [17,19,20] reported no significant associations, other studies [18,21,22] revealed a negative association with the eating behaviors of children. For example, a study conducted in Ireland by Bassul et al. (2020) [18] found that children whose caregivers pressured them to eat were 33% less likely to consume fruit daily. This suggests that pressuring children may discourage them from adopting healthy eating habits, potentially due to increased resistance or negative associations with certain foods.

Instrumental Feeding (Using Food as a Reward). Instrumental feeding, where food is used as a reward, emerged as a key contributor to unhealthy eating behaviors in children. Instrumental feeding practices were linked to less favorable eating behaviors in children. While some studies found no significant associations [20,24], others highlighted negative impacts. Berge et al. (2020) [17] noted a bidirectional relationship where children’s high food responsiveness led parents to use food as a reward, further reinforcing the child’s responsiveness and potentially fostering unhealthy eating cycles, especially in children between 2 and 4 years old. On the other hand, a study that we reviewed indicated that using food as a reward might be to enhance the consumption of traditional food. In China, for example, Xiang et al. (2021) [22] found that, especially among normal-weight children, the use of food as a reward by parents was negatively associated with the adherence to traditional dietary patterns. In other words, parents who frequently use food as a reward tend to have children who are less aligned with healthier, traditional eating habits [22]. These findings indicate that using food as a reward may undermine healthy eating habits and promote preferences for less nutritious foods.

Modeling, Teaching, and Encouragement of Healthy Eating. Parental modeling involves demonstrating healthy eating behaviors that children can imitate, whereas encouragement refers to the active promotion and support of healthy eating habits in children. Parental modeling and encouragement were generally associated with positive eating behaviors in children. Quah et al. (2018) [20] revealed that mothers who modeled healthy food intake had children who consumed significantly more vegetables, whole grains, and less unhealthy snacks and fast food. The study has also reported that encouraging balance and variety in the child’s diet was associated with children eating more vegetables by 19.5 g/day. Additionally, the study revealed that teaching children about nutrition was linked to more whole grain consumption by 9.41 g/day [20]. However, Berge et al. (2020) [17] reported that over time, encouragement did not significantly change children’s eating behaviors, suggesting that consistent and possibly more active strategies may be necessary to sustain healthy habits.

Monitoring. Monitoring refers to parents’ supervision of their children’s eating habits, including keeping track of the types and amounts of food consumed. Parental monitoring showed varying effects on children’s eating behaviors. While some studies revealed no impact [21,24], other studies [22,23] found positive associations with healthier eating patterns. Xiang et al. (2021), [22] for example, found that parental monitoring was linked with lower snacking dietary patterns and higher traditional dietary patterns across weight groups. This indicates that active parental involvement in monitoring can reduce the intake of unhealthy snacks and promote the consumption of healthier, traditional foods [22]. Additionally, Warkentin et al. (2018) [23] reported that parents who engaged in less monitoring had children who consumed more ultra-processed foods and spent more than two hours a day in front of screens. Overall, this variation in findings highlights the need for further research to better understand the conditions under which caregiver monitoring is most effective.

Emotion Feeding Practice. Emotional feeding practices refer to parental behaviors where food is used to manage a child’s emotional states, either by offering food as a reward or to soothe negative emotions. The use of food to manage children’s emotions yielded mixed results. Berge et al. (2020) [17] conducted a longitudinal observational study in the United States to examine bidirectional relationships between parental feeding practices, including emotional feeding, and child eating behaviors among preschool-aged children from low-income households. The study found that emotional feeding predicted initial increases in satiety responsiveness in children, but this effect diminished over time. The practice may lead children to rely on external cues rather than internal hunger signals, potentially promoting overeating [17]. In contrast, other studies [19,20] found no significant associations between emotional feeding and children’s eating behaviors. For instance, Quah et al. (2018) reported no significant associations between emotional feeding practices and the intake of fruits, vegetables, whole grains, or unhealthy foods among preschoolers [20]. Similarly, in the United Kingdom, Haycraft et al. (2017) [19] explored how feeding practices differed between mothers of healthy weight and those with overweight or obesity. While the study primarily focused on various controlling feeding practices, it found no significant differences between the two groups in the use of food for emotion regulation [19]. This suggests that the impact of emotional feeding may vary based on other moderating factors such as cultural context or individual child differences.

Permissive Feeding Practices. Permissive feeding practices, where parents give children more autonomy in food choices, often lead to negative dietary outcomes. Despite the limited studies in this review examining the impact of parental feeding control, permissive feeding was consistently associated with negative dietary outcomes. Overall, the results showed that allowing children to have uncontrolled autonomy over food choices often led to less healthy eating behaviors.

Lindsay et al. (2018) [16], for example, reported that many low-income Latino immigrant parents exhibited permissive feeding styles, allowing children to choose unhealthy foods and engage in excessive screen time. This was often due to logistical and socioeconomic challenges that made it difficult for parents to consistently enforce healthy eating behaviors at home [16]. Additionally, Quah et al. (2018) [20] found that allowing a child to have control over food choices was associated with a lower vegetable and whole grain intake and a higher consumption of sweet snacks and fast food. Similarly, Lindsay et al. (2020) [15] found that Brazilian immigrant fathers were more permissive in their feeding practices, allowing their children to consume sweets and high-calorie foods. Fathers used food as a reward and felt that such practices were part of childhood, but this permissiveness conflicted with their role as healthy eating role models [15]. Haycraft et al. (2017) [19] found that mothers with overweight or obesity were more likely to give their children greater control over their food choices, which resulted in a less balanced and varied diet, as well as a less healthy home food environment. In conclusion, these studies suggest that permissive feeding practices may undermine efforts to establish healthy eating habits in children, particularly when combined with external stressors like socioeconomic challenges.

Overall, the findings highlight key factors influencing preschoolers’ dietary behaviors, as summarized in Table 1, which provides an overview of these factors and their associations with eating habits.

## 4. Discussion

Guided by the construct of the TPB, this review sought to synthesize the existing literature on the association between the eating behaviors of preschool children aged 2 to 5 years and one or more of the following predictors: personal and household characteristics, primary caregiver dietary beliefs, intentions to provide a healthy diet, or feeding practices.

### 4.1. Personal and Household Characteristics

Through the studies reviewed, we identified several personal and household characteristics that significantly influenced preschoolers’ eating behaviors. These factors included weight perceptions, concerns about weight, desired weight, food availability, food accessibility, and television viewing habits.

Despite the limited evidence, the studies reviewed have shown a significant association between the perception and concerns of caregivers about their children’s weight and their children’s eating behaviors. This association was either direct or mediated through the feeding practices of caregivers. A study by Xiang et al. (2021) [22], for example, highlighted that parental concerns about child overweight risk were associated with specific feeding practices, such as restriction and pressure to eat, which subsequently influenced children’s eating behaviors [22]. These results align significantly with current studies showing a significant association between parental perception of their child’s weight and their feeding practices. Studies found that parents who perceive their child to be overweight or obese were more likely to use authoritarian feeding styles, such as control and restrictive practices [31,32]. Contrary to these findings, the study by Kutbi and Mosli (2024) [24] revealed a direct association between caregiver perceptions and concerns regarding their child’s weight and several key eating behavior constructs, including food responsiveness, satiety responsiveness, and food fussiness. No association was found with feeding practices, such as restriction and monitoring [24]. This discrepancy in findings highlights the complexity of the relationship between caregiver perceptions, feeding practices, and children’s eating behaviors. These contrasting results suggest that additional factors may influence this relationship. Variables such as cultural context, socioeconomic status, or individual child characteristics might play a significant role. It underscores the need for further research to explore these associations comprehensively and to identify potential moderating or mediating factors.

This review has revealed a consistent and significant relationship between preschoolers’ eating behaviors and their primary caregiver’s BMI and the food environment at home (e.g., food availability and accessibility) [18,19]. These findings align with the existing literature indicating that parental dietary behaviors and weight status can significantly influence children’s eating habits through modeling and the home food environment [3,33]. A global study, for example, reported that preschoolers were more likely to consume sugary foods if such items were readily available at home, even in the presence of healthier options like fruits and vegetables [34]. Furthermore, TV viewing was associated with lower consumption of fruits and vegetables [18], highlighting findings from a systematic review of 19 studies that linked increased TV viewing time with a higher consumption of unhealthy, calorie-dense foods [35]. These findings suggest that screen time not only displaces healthy eating opportunities, but also exposes children to unhealthy food advertising, which could drive poor dietary choices.

However, personal characteristics such as sex, race/ethnicity, and marital status were not thoroughly examined in the reviewed studies for their direct impact on preschoolers’ eating behaviors. Instead, these factors were typically treated as covariates or background variables. A deeper understanding of their influence could offer valuable insights into the factors shaping preschoolers’ dietary habits and help tailor strategies to promote healthier lifestyles across diverse populations.

### 4.2. Dietary Beliefs and Intentions to Provide a Healthy Diet

The evidence was limited in addressing the impact of dietary beliefs and intentions to provide a healthy diet on the eating behaviors of preschoolers. Two qualitative studies found that while caregivers understood the importance of healthy eating and aimed to model good habits for their children, they faced challenges that limited their ability to effectively promote and maintain a healthy diet for their children [15,16]. These challenges included conflicting work schedules, financial limitations, and neighborhood safety concerns, leading to more permissive feeding practices and allowing children greater control over their food choices, which negatively affected their eating behaviors.

Prior studies have focused on exploring the impact of caregivers’ dietary knowledge on the eating behaviors of children. The studies found a positive association between caregivers’ dietary knowledge and the eating behavior of children [36,37,38]. These findings suggest that enhancing caregivers’ dietary knowledge could be a key intervention for improving preschoolers’ diet quality. However, no studies have quantitatively examined the direct or indirect effects of caregivers’ dietary beliefs or intentions on preschoolers’ eating behaviors. Understanding this relationship could guide targeted interventions that address caregivers’ beliefs and motivations, leading to more effective feeding practices.

### 4.3. Feeding Practices

This review comprehensively examines how various parental feeding practices influence the eating behaviors of preschoolers. The results revealed a complex interplay between caregiver strategies and child dietary outcomes, with practices like monitoring, modeling, teaching, and encouraging healthy eating generally showing positive impacts. This aligns with prior research suggesting that positive parental involvement can encourage children to adopt healthier dietary habits, such as a higher consumption of fruits and vegetables [3,6,39]. Including this review, the existing studies showed that when parents model healthy eating behaviors, children are more likely to eat nutritious foods [40,41]. This influence appears to persist from childhood through adolescence and even into early adulthood.

This review has also found that feeding practices, including using food as a reward (instrumental feeding), pressure to eat, emotion, permissive, and certain forms of restriction, were associated with less favorable dietary outcomes in preschoolers. These findings align with and extend the current literature, highlighting both consistencies and discrepancies that deepen our understanding of this important aspect of child development. For instance, restrictive feeding practices emerged as having mixed effects on children’s eating behaviors. In some contexts, such as in Singapore and China, the restriction on feeding practices was linked to increased fruit intake and reduced snacking behaviors, respectively [20,22]. Conversely, in Ethiopia, restriction was associated with increased food responsiveness and emotional overeating in children [21]. A similar variability is noted in the broader literature. Birch et al. (2003) [42] found that restriction can lead to an increased desire for restricted foods. This can potentially lead to overeating when those foods become available (a phenomenon known as the “forbidden fruit effect”). On the other hand, Vollmer and Mobley (2013) suggest that moderate restriction, when appropriately applied, can help in reducing the intake of unhealthy foods without negative repercussions [43]. Additionally, the existing studies revealed that covert restriction (subtle limitation without overt control) is associated with a higher diet quality compared to overt restriction (direct and noticeable control) [44,45]. Additionally, the pressure to eat has shown a mixed effect on the eating behaviors of preschool children. For instance, some studies reviewed that the pressure to eat had a null association [17,19,20], while other studies revealed a significant negative association with the eating behaviors of children [18,21,22]. This inconsistent impact aligns with the existing literature showing both significant associations and no associations between the pressure to eat and children’s eating behaviors. A systematic review and meta-analysis by Yee, Lwin, and Ho (2017) [41] analyzed 22 studies on the relationship between the pressure to eat and healthy food consumption. The findings revealed that 14 studies showed no significant associations, 6 reported significant negative associations, and only 2 found positive associations. Regarding unhealthy food consumption, the review found that 8 studies identified a significant positive association, while 13 showed no association [41].

Other feeding practices, including using food as a reward (instrumental feeding) and emotion, have also shown mixed effects on the eating behaviors of children. This aligns with the existing literature, which has found either no association or significant negative associations with the eating behaviors of children [41,46,47,48]. These studies indicated that the impact of emotional feeding may be more pronounced in younger children, and, over time, it could contribute to the development of unhealthy eating behaviors, such as fussiness. Overall, the findings appear to be complex and may depend on several factors, such as the child’s age, gender, individual characteristics, and the length of applying such a feeding practice.

Largely consistent with the findings of this review, other studies have shown that permissive feeding approaches, characterized by low control or demandingness regarding children’s food choices and intake, were associated with less healthy dietary patterns in children. This includes a lower consumption of nutrient-dense foods and a higher intake of low-nutrient-dense or unhealthy foods. Additionally, permissive feeding styles have been linked with a higher BMI in children [41,49,50,51].

Overall, the literature reveals significant gaps, including the limited investigation into the direct effects of caregivers’ beliefs and intentions on children’s eating behaviors and the insufficient consideration of cultural and socioeconomic factors. Furthermore, the inconsistent findings regarding the impact of feeding practices, such as restriction and the pressure to eat, highlight the necessity for standardized definitions and comprehensive contextual analyses. These gaps hinder the ability to draw robust and generalizable conclusions.

### 4.4. Strengths and Limitations

To our knowledge, this is the first integrative review using the TPB to comprehensively synthesize the relationship between caregivers’ dietary beliefs, intentions, and feeding practices with preschoolers’ eating behaviors. However, this review has several limitations. The included studies were published between 2014 and 2024, with most of the research conducted in high-income countries, which may limit the generalizability of the findings to other cultural and socioeconomic contexts. Furthermore, the exclusion of non-English, unpublished, and gray literature may have introduced publication bias, further constraining the generalizability of the results. Additionally, the lack of a previously published protocol represents a limitation of this review, as it may impact the transparency and replicability of the methodology. The use of database filters, while necessary to refine the search results, may have inadvertently excluded relevant studies, potentially affecting the comprehensiveness of the findings.

Finally, while our review included studies with varied designs, studies in this review did not quantitatively examine the direct relationships between caregivers’ beliefs, intentions, and children’s eating behaviors. Most studies relied on cross-sectional designs, limiting causal conclusions and contributing to inconsistent findings, particularly regarding the impact of feeding practices.

## 5. Conclusions

This integrative review highlights the critical influence of caregivers’ dietary beliefs, intentions, and feeding practices on the eating behaviors of preschool-aged children, using the Theory of Planned Behavior (TPB) as a guiding framework. Positive practices like modeling, teaching, monitoring, and encouraging healthy eating are consistently associated with favorable dietary outcomes, while practices such as using food as a reward, emotional feeding, and permissive styles often yield less favorable results.

Future research should prioritize longitudinal designs to establish causal relationships, address the gaps in the limited available literature, and explore broader socioecological influences. Developing culturally sensitive interventions targeting caregivers’ beliefs and practices is also critical. These efforts can help foster healthy eating behaviors during the preschool years, thereby promoting long-term health outcomes.

## Figures and Tables

**Table 1:** Summary of evidence on factors associated with the dietary behaviors of preschoolers.

Factors	Key Findings	Studies
Qualitative Studies		
Behavioral Beliefs	While some studies mention that caregivers are aware of the benefits of healthy eating, there is limited quantitative research directly exploring the association between caregivers’ behavioral beliefs and preschoolers’ eating behaviors.	[15,16]
Normative beliefs	Some studies explore cultural or social expectations (normative beliefs), but there is limited quantitative evidence directly linking normative beliefs with the eating behaviors of preschoolers.	[15]
Control beliefs	Studies did not directly explore the association between caregivers’ control beliefs and their children’s eating behaviors, though control factors like finances and environment are mentioned as barriers in some studies.	[16]
Intentions to Provide a Healthy Diet	Low-income Latino immigrant caregivers aimed to provide healthy diets but faced barriers like financial constraints, conflicting work schedules, and neighborhood safety concerns. Brazilian immigrant caregivers intended to provide healthy food and teach their children about nutrition but encountered cultural and logistical challenges in doing so consistently. Studies do not directly examine the relationship between these intentions and preschoolers’ actual eating behaviors.	[15,16]
**Quantitative Studies**		
**Personal Characteristics**		
Weight perception and concern	Primary caregivers’ perceptions and concerns about child weight were significantly associated with their preschoolers’ dietary behaviors. Misperceptions led to practices like pressure to eat or restriction.	[22,24]
Caregivers’ BMI	Higher caregiver BMI was associated with less healthy dietary behaviors in preschoolers, such as a lower fruit and vegetable intake and a higher consumption of sweets and sugary beverages.	[18,19]
**Household Characteristics**		
Food environment	The availability of fruits and vegetables at home was positively linked to preschoolers’ higher fruit and vegetable intake, while the availability of sugary snacks was linked to the increased consumption of unhealthy foods.	[18]
TV viewing	TV viewing was inversely associated with healthy eating behaviors in preschoolers, such as a lower vegetable intake and a higher consumption of snacks and sugar-sweetened beverages (SSBs).	
**Feeding Practices**		
Restrictive feeding	Restrictive feeding practices showed mixed outcomes: in Brazil, restriction was linked to weight concerns but not to eating behavior. In Ethiopia, it increased food responsiveness and emotional overeating but decreased food fussiness. In Singapore and China, it was associated with increased fruit intake and reduced snacking.	[20,21,22,23]
Pressure to eat	Pressuring children to eat was associated with negative outcomes such as a lower fruit intake and increased snacking. In Ireland, children whose caregivers applied pressure to eat were 33% less likely to consume fruits. Some studies found no significant associations.	[17,18,19,20,21,22,23]
Instrumental feeding (reward-based)	Using food as a reward was linked to less favorable eating behaviors, such as reinforcing food responsiveness and unhealthy eating cycles. In China, using food as a reward was negatively associated with the adherence to traditional dietary patterns, particularly in normal-weight children.	[17,20,22,24]
Modeling, Teaching, and Encouragement of Healthy Eating	Parental modeling and encouragement of healthy eating were generally associated with positive behaviors, such as an increased vegetable and whole grain consumption. However, encouragement alone did not always sustain long-term changes in eating habits.	[17,20]
Monitoring	Parental monitoring showed mixed results: it was linked to lower snacking and higher traditional dietary patterns in China, but some studies reported no significant associations with children’s eating behaviors. Less monitoring was linked to a higher consumption of ultra-processed foods.	[21,22,23,24]
Emotion	Emotional feeding had mixed results. In some studies, it was linked to increased satiety response in children, but over time, this effect diminished, potentially promoting overeating. Other studies found no significant associations between emotional feeding and eating behaviors.	[17,19,20]
Permissive feeding	Permissive feeding, where parents give children autonomy over food choices, was consistently associated with negative dietary outcomes, including a lower vegetable intake and a higher consumption of sweet snacks and fast food. This was often due to external stressors like socioeconomic challenges.	[15,16,19,20]

## Data Availability

Not applicable.

## Supplementary Materials

The following supporting information can be downloaded at: https://www.mdpi.com/article/10.3390/children12020163/s1, Table S1: Summary of Research Findings Associated with the Eating Behaviors of Children.
