# Supplementary material for: Understanding Caregivers’ Influence on Preschoolers’ Eating Behaviors: An Integrative Review Guided by the Theory of Planned Behavior
Source: Children (Basel). 2025 Jan 29;12(2):163. doi: 10.3390/children12020163 (PMC11854435; doi:10.3390/children12020163)
Supplement: Supplementary file 1 [file children-12-00163-s001.zip › children-3419676-supplementary.pdf]

Supplemental Table S1. Summary of Research Findings Associated with the Eating Behaviors of Children.

Ref/ Country/ Design/ Theory	Purpose/ Sample Characteristics / Setting	Outcome Measures	Key Findings	Level of Evidence (LOE)* and Main Limitations
[18]<br><br>Ireland<br><br>Cross-Sectional Study<br><br>Theory not stated	**Purpose:** To investigate the relationship between home environmental characteristics (parental control feeding practices, family mealtimes, food availability, etc.) and preschool children's intakes of fruits, vegetables, and confectionery/sugar-sweetened beverages (SSBs).<br><br>**Sample Characteristics:** 332 preschool children (aged 3-5 years) and their parents/guardians. Parents were mostly mothers (88.3%), predominantly Irish (66.9%), and aged 30-39 years (63.6%).<br><br>**Setting:** Preschool children from Dublin, Ireland, recruited through randomized stratified sampling.	• Children's and Parents' Food Consumption using Confectionery/Sugar-Sweetened Beverages (SSBs).	• Higher education levels in parents were associated with children's higher intake of fruits and vegetables and lower intake of confectionary/SSBs.<br>• Children whose parents were overweight or obese were less likely to consume fruits and vegetables and more likely to consume confectionary/SSBs.<br>• Pressure to eat was negatively associated with children's fruit intake. Children whose parents applied more pressure to eat were 33% less likely to consume fruits daily.<br>• Watching more than 1 hour of television per day was associated with lower vegetable intake and higher confectionary/SSB intake.<br>• Children who ate snacks while watching television were 71% less likely to consume vegetables daily compared to those who did not.<br>• The availability of fruits and vegetables in the home was positively associated with children's fruit and vegetable consumption.<br>• The availability of sugary snacks and SSBs in the home was positively associated with children's intake of these foods.	• **LOE: III**<br><br>**Limitations:**<br>• Cross-sectional design limits causal interpretation.<br>• Self-reported data may lead to recall bias or positive response bias.<br>• The study sample may not be nationally representative, limiting the generalizability of the findings.<br>• The conceptual definition of the main concepts was not clearly defined.<br>• No theoretical framework.

[17]<br><br>USA<br><br>Longitudinal observational study (secondary analysis of data from the NET-Works randomized controlled trial).<br><br>Theory not stated	**Purpose:** To examine bidirectional relationships between parental feeding practices (e.g., instrumental, emotional, control, and encouragement) and child eating behaviors (e.g., food responsiveness, satiety response, food fussiness, and enjoyment of food) in preschool-aged children**.**<br><br>**Sample Characteristics:** 534 children aged 2–4 years, above the 50th percentile for height and weight, from low-income households (<$65,000 annual income). The majority of children were Hispanic (57.8%) and from families with varying levels of education and income.<br><br>**Setting:** Households in Minneapolis and St. Paul, Minnesota, with in-home measurements taken across four time points (baseline, 12, 24, and 36 months).	• Child Eating Behaviors: Measured using the Children's Eating Behaviour Questionnaire (CEBQ).	• Parental instrumental feeding and child food responsiveness showed strong bidirectional associations over time. For instance, a child's high food responsiveness could lead to more instrumental feeding by the parent (e.g., using food as a reward), and the use of instrumental feeding could further enhance food responsiveness in children.<br>• Parental emotional feeding was linked with both child satiety response and child food responsiveness, showing a unidirectional influence where emotional feeding practices (e.g., feeding based on emotions) predicted changes in child eating behaviors over time.<br>• Instrumental feeding: Using food as a reward/bribe reinforced high food responsiveness in children, leading to a feedback loop where the child's responsiveness promotes more instrumental feeding from parents.<br>• Emotional feeding: Initially, emotional feeding was associated with higher satiety response in children, but over time, it led to a decrease in satiety response, suggesting a more complex dynamic as the child grows older.<br>• Other parental feeding practices like pressure to eat or encouragement to eat were not as strongly associated with significant changes in child eating behaviors over time, compared to instrumental and emotional feeding.	• **LOE**: III<br><br>**Limitations:**<br>• The study included only children from the 50th BMI percentile and above, limiting the generalizability to children with lower BMI.<br>• Certain confounding variables, such as child temperament, were not assessed, which could have influenced the findings.<br>• Modest explanatory power of the cross-lagged models, suggesting that additional factors may contribute to the relationships between parental feeding practices and child eating behaviors.

[16]<br><br>US<br><br>Qualitative Study using Focus Group Discussions (FGDs)<br><br>Health-Promoting Family Framework and Social Cognitive Theory	**Purpose:** To explore low-income Latino immigrant parents' beliefs, parenting styles, and practices related to their preschool children's eating, physical activity (PA), and sedentary behaviors (SB) at home to identify intervention strategies.<br><br>**Sample**: 33 participants (27 mothers, 6 fathers), average age 29 ± 2.3, low-income Latino immigrant parents of children aged 2-5 years, recruited through family childcare homes (FCCHs) in Massachusetts.<br><br>**Setting**: Licensed Latino FCCHs and public library meeting rooms.	Recruited parents were asked to answer questions about 1) parents' beliefs and attitudes related to eating, physical activity, and sedentary behaviors, 2) parents' perceptions of children's eating and physical activity experiences at home, 3) parenting practices related to eating, 4) physical activity and sedentary behaviors at home, and 5) barriers faced in structuring a home environment conducive to healthy eating and physical activity.	• Parents recognized the importance of healthy eating and PA for their children's growth but reported challenges in maintaining these habits at home.<br>• Parents aimed to be role models but struggled with their own unhealthy eating habits and lack of PA, often due to time pressures, financial constraints, and lack of space.<br>• Socioeconomic and logistical challenges, such as conflicting work schedules, financial limitations, neighborhood safety concerns, and screen time limits, made it difficult for parents to support healthy eating and PA behaviors in their children.<br>• Many parents exhibited a permissive parenting style, often allowing unhealthy food choices and excessive screen time.	• **LOE**: III<br><br>**Limitations:**<br>• The study had a small sample size and used purposive sampling, limiting generalizability.<br>• The study is focused on Latino families in Massachusetts, which may not represent all low-income Latino families across the U.S.<br>• Participants may have self-selected into the study due to an interest in healthy behaviors, and responses may be influenced by social desirability bias.

[15]<br><br>USA<br><br>Exploratory descriptive qualitative study.<br><br>Socio-ecological model and social contextual model.	**Purpose:** To explore the perspectives and practices of Brazilian immigrant fathers regarding their preschool-aged children's eating and feeding habits.<br><br>**Sample:** 21 Brazilian immigrant fathers aged 27–43 years, living in Massachusetts for an average of 7.3 years, with at least one child aged 2–5 years.<br><br>**Setting:** Fathers were recruited through Brazilian businesses, community organizations, and social services, as well as network and snowball sampling methods.	Recruited parents were asked to answer four questions 1) definitions of healthy eating, 2) beliefs and attitudes relating to young children's healthy Eating, 3) practices relating to children's eating, and 4) barriers to children's healthy eating.	• Fathers viewed food as central to Brazilian culture and saw family meals as an opportunity to teach children about healthy eating and Brazilian customs.<br>• The home food environment, including the availability of traditional Brazilian food and the restriction of unhealthy food, played a key role in shaping children's eating habits.<br>• Fathers used a permissive feeding style, often allowing children to eat sweets and high-calorie foods, citing it as part of childhood. They also used food as a reward and showed concern about children eating unhealthy foods outside the home.<br>• Fathers felt they had a role in modeling healthy eating for their children but recognized that mothers were the primary decision-makers in feeding practices.<br>• Fathers expressed interest in learning more about healthy eating for their children and were open to participating in interventions to promote healthy eating.	• **LOE: III**<br><br>**Limitations:**<br>• Small, purposive sample that may have a selection bias, as fathers who participated might have had a particular interest in the study's topic.<br>• Findings are not generalizable beyond the specific group of Brazilian immigrant fathers in Massachusetts.<br>• The study relied on self-reported data from fathers, which may be influenced by social desirability bias.

[19]<br><br>UK<br><br>Cross-sectional study<br><br>Theory not stated	**Purpose:** To investigate how feeding practices and perceptions of child eating behaviors differ between mothers with healthy weight and those with overweight/obesity.<br><br>**Sample Characteristics:** 437 mothers (mean age 34) with children aged 2-6 years, recruited through schools, nurseries, and social media across the UK.<br><br>**Setting:** UK-based families with young children, predominantly White British, and relatively high levels of education (51% had university degrees or higher).	• Children's Eating Behavior Questionnaire (CEBQ), assessing child behaviors such as food responsiveness, satiety responsiveness, desire to drink, and more.	• Mothers with overweight/obesity were more likely to:<br>• Give their child more control overeating.<br>• Encourage less balance and variety in the diet.<br>• Have a less healthy home food environment.<br>• Model less healthy eating behaviors.<br>• Mothers with overweight/obesity perceived their children as having a higher desire for drinks, being more responsive to satiety, and eating more slowly compared to children of mothers with healthy weight.<br>• No significant differences between the groups in the use of controlling feeding practices such as pressure to eat or using food for emotion regulation.	**LOE**: III<br><br>**Limitations:**<br>• Self-reported height and weight data may introduce bias.<br>• The sample was predominantly White British and highly educated, limiting generalizability.<br>• Multiple analyses without correction for potential false positives due to repeated testing.<br>• Small effect sizes, indicating that the differences between groups might not be large in practical terms.

[20]<br><br>Singapore<br><br>Cross-sectional study within the Growing Up in Singapore Toward healthy Outcomes (GUSTO) Study.<br><br>Theory not stated	**Purpose:** To investigate how 12 maternal feeding practices, captured by the CFPQ, relate to dietary intakes and BMI of 5-year-old preschoolers in Singapore.<br><br>**Sample Characteristics:** 511 mother-child pairs from a multi-ethnic population (Chinese, Malay, Indian) in Singapore. The mothers had a mean age of 30 years, and about 70% had post-secondary education.<br><br>**Setting:** Singapore, with participants recruited from public maternity hospitals.	• Dietary intake at 5 years of age. Food Frequency Questionnaire (FFQ), 112 food items.	• Children of mothers who modeled healthy food intakes consumed significantly more vegetables (20.0g/day) and more wholegrains (20.9g/day) compared to those whose mothers did not model healthy eating behaviors.<br>• Encouraging balance and variety in the child's diet was associated with children eating 19.5g/day more vegetables.<br>• Teaching children about nutrition was linked to 9.41g/day more wholegrain consumption.<br>• Restricting foods for weight control was associated with children consuming 28.5g/day more fruit.<br>• Children of mothers who allowed more child control over food intake (i.e., lack of parental control) consumed 15.2g/day less vegetables and 13.6g/day less wholegrains.<br>• Mothers who modeled healthy food intakes had children who consumed 10.1g/day less sweet snacks and 5.84g/day less fast food.<br>• Mothers who allowed more child control had children who consumed 13.7g/day more sweet snacks and 6.63g/day more fast food.<br>• No significant associations were found between other feeding practices (e.g., using food as a reward, food restrictions for health, emotional regulation, or pressure to eat) and the intake of fruits, vegetables, wholegrains, sugar-sweetened beverages, sweet snacks, fast foods, or fried foods.	**LOE: III**<br><br>**Limitations:**<br>• Cross-sectional design limits causal inferences and may introduce reverse causality.<br>• Self-reported data on feeding practices and dietary intakes may introduce social desirability bias and misclassification.<br>• Generalizability may be limited as the study focused on highly educated mothers and children aged 5, *which* may not represent the broader population or older children.

[21]<br><br>Ethiopia<br><br>Cross-sectional, school-based study<br><br>Theory not stated	**Purpose:** To examine the associations between caregivers' feeding practices and eating behaviors among preschool children in Addis Ababa.<br><br>**Sample Characteristics:**<br>Participants: 525 caregiver-child pairs (children aged 3-6 years).<br>Caregivers: Mostly mothers (92.2%), with 63.7% having secondary education or higher.<br>Children: Mean age of 4.5 years, with 47% male and 53% female.<br><br>**Setting:** Preschools in Addis Ababa, Ethiopia, stratified based on socioeconomic status.	• Children's Eating Behaviors – Assessed using the Children's Eating Behaviour Questionnaire (CEBQ).	• Caregivers' practice of food restriction was positively associated with enjoyment of food, food responsiveness, emotional overeating, and desire to drink.<br>• Caregivers' practice of pressure to eat was negatively associated with enjoyment of food, food responsiveness, and emotional overeating.<br>• Caregivers' Food Restriction was negatively associated with food fussiness, but positively associated with emotional undereating.<br>• Caregivers' pressure to Eat was positively associated with food fussiness, Satiety responsiveness, and slowness in eating.<br>• The practice of monitoring was not significantly associated with any of the children's eating behaviors.	**LOE**: III<br><br>**Limitations:**<br>• Cross-sectional design limits the ability to infer causal relationships between feeding practices and eating behaviors.<br>• Self-reported measures may introduce biases as caregivers might not accurately report their child's behaviors or their own feeding practices.<br>• The CEBQ and CFQ were not validated in the Ethiopian context, which might affect the accuracy of the measurements.<br>• Limited generalizability as the study only included preschoolers from urban areas of Addis Ababa, making it difficult to generalize findings to rural populations.

[22]<br><br>China<br><br>Cross-sectional study<br><br>Theory not stated.	**Purpose:** To examine the interrelationships between parental child weight perceptions (misperception, concern, dissatisfaction), feeding practices, and children's dietary intake among Chinese preschoolers and their parents.<br><br>**Sample Characteristics:** 1,616 parent-child pairs (53.7% boys), with preschool children from Changsha City, Hunan Province. The average age of children was 4.54 years, and 88.8% of the parents were mothers.<br><br>**Setting:** Six public kindergartens in Changsha City, Hunan Province, China (January to March 2021).	• Children's dietary patterns (traditional vs. snacking) using a 27-item food frequency questionnaire (FFQ).	• Parental restriction was inversely associated with snacking dietary patterns across weight groups, but no association with traditional dietary patterns.<br>• Food as Reward was negatively associated with traditional dietary patterns in normal-weight children.<br>• Pressure to eat was associated with higher snacking dietary patterns in normal-weight children, but negatively associated with traditional dietary patterns in underweight children.<br>• Monitoring was linked negatively with snacking dietary patterns in underweight and normal-weight children, and positively associated with traditional dietary patterns across all weight groups.<br>• Parents who underestimated their child's weight applied more pressure to eat in normal-weight children and overweight/obese children.<br>• Parents concerned about their child becoming overweight applied greater restrictions for normal-weight children.<br>• For underweight children, parents who wished for a thinner child reported a higher snacking dietary pattern score.<br>• Overestimating child weight was linked to a higher traditional dietary pattern score in underweight children.	**LOE**: III<br><br>**Limitations:**<br>• Cross-sectional Design: This design does not allow for causal inferences between parental perceptions, feeding practices, and children's dietary patterns.<br>• Self-reported Data: Children's height and weight were self-reported by parents, which may introduce recall bias.<br>• Limited Generalizability: The study was conducted in a single city (Changsha), limiting its generalizability to other regions of China or globally.

[23]<br><br>Brazil<br><br>Cross-sectional study<br><br>Theory not stated.	**Purpose:** To investigate the relationships between parental feeding practices, parental attitudes toward child weight, child weight status, and children's consumption of ultra-processed and traditional foods.<br><br>**Sample Characteristics:** The final sample includes 402 parent-child pairs. The vast majority of questionnaires were completed by mothers (93.54%). The majority of parents were highly educated, with 90% having college-level education or higher.<br><br>**Setting:** 14 schools participated, with questionnaires distributed to parents of children attending these schools.	• Children's eating behaviors: Evaluated using the Food Frequency Questionnaire (FFQ).	• Restriction for Weight Control was more common among parents with lower maternal education and higher concern about child overweight.<br>• Restriction for Health was associated with lower maternal education.<br>• Parents who applied more pressure to eat were 2.3 times more likely to be concerned about child underweight, and this was linked to lower maternal BMI.<br>• Greater use of Emotion Regulation/Food as Reward was associated with lower maternal education.<br>• Lesser use of Healthy Eating Guidance was linked to:<br>• Infrequent consumption of traditional foods.<br>• Increased screen time of more than two hours per day.<br>• Higher maternal BMI.<br>• Lesser use of Monitoring was linked to:<br>• Greater consumption of ultra-processed foods.<br>• More than two hours of screen time per day.<br>• Lower perceived responsibility for child feeding.	**LOE**: III<br><br>**Limitations**:<br>• The study relies on self-reported data for both parental feeding practices and children's dietary intake, which may introduce bias.<br>• Parental perceptions of child weight were subjective and may not accurately reflect children's true health status.<br>• The sample is predominantly mothers, limiting the ability to generalize findings to fathers or other caregivers.<br>• The cross-sectional design does not allow for causal inferences.

[24]<br><br>Saudi Arabia<br><br>Cross-sectional study	**Purpose**: To examine the associations of maternal concern and perception about child overweight risk with maternal feeding practices and child eating behaviors among Saudi preschoolers aged 3-5 years old.<br><br>**Sample Characteristics**. The sample consisted of 115 mother-child pairs recruited from eight preschools across different regions of Jeddah, Saudi Arabia.<br>Majority of the children (59.1%) were boys, and most mothers had completed a college degree or higher (80%).<br><br>**Setting.** Eight preschools are located in different regions of Jeddah, Saudi Arabia. These preschools were used as the sites for recruiting mother-child pairs for participation in the study.	• Child eating behaviors (CEBQ): Assessed using constructs like enjoyment of food, food responsiveness, satiety responsiveness, food fussiness, and slowness in eating.	• Maternal concern about child weight was positively correlated with child enjoyment of food and child food responsiveness.<br>• No significant correlations were found between maternal concern about child weight and feeding practices, such as restriction, using food as a reward, or monitoring.<br>• Maternal perception of child overweight risk was positively correlated with child BMI-z score and negatively correlated with child slowness in eating.<br>• Maternal concern was positively associated with child enjoyment of food and child food responsiveness, but negatively associated with child food fussiness.<br>• Maternal perception of child overweight risk was inversely related to child slowness in eating.<br>• No significant associations were found between maternal concern or perception about child overweight risk and maternal feeding practices, including the use of restriction, food as a reward, or monitoring.	**LOE: III**<br><br>**Limitations:**<br><br>• The sample size of 115 mother-child pairs was relatively small, and the response rate was 55%, which may limit generalizability.<br>• The study design does not allow for causal inferences.<br>• Maternal height and weight were self-reported, which could introduce recall bias.<br>• The high proportion of mothers with a college degree may limit the generalizability to less educated populations.

* **LOE**: Johns Hopkins Nursing Evidence-Based Practice appraisal.
